# The Administration of Chloroform

**Published:** 1882-09

**Authors:** 


### THE ADMINISTRATION OF CHLOROFORM.

The *Gazette des Hopitaux*, at the end of the *resume* of the prolonged discussion on this subject which has just terminated at the Academie de Medecine, furnishes the following account of the rules of precedure observed by a *collaborateur* who has been much employed with constant success, in the administration of chloroform during the last ten years:

1. The compress is to be preferred to all other means. A handkerchief is to be had every where, and alarms the patient less than anything else.

2. Fold the handkerchief into the form of the mouth of a horn, and keep it closely pressed against the point of the nose; but only pour the chloroform on the part of it which is not directly in contact with the skin.

3. Its application should be intermitted, but this need not be done in the precisely regulated manner recommended by Professor Gosselin.

4. Give very little chloroform at the commencement, in order to accustom the patient to it and prepare him for the feeling of suffocation. Then, when the first inspirations are over, pour on the chloroform very often, otherwise much time will be lost and complete anesthesia obtained only with difficulty.

5. Before making the application take care that no article of dress constricts the patient, removing even the string of a cap.

6. Expose the epigastrium, and from the very commencement keep the eye upon it, and constantly watch the respiration, without caring about the pulse.

7. Always have a forceps within reach.

8. As soon as the respiration becomes noisy and stentorous, remove the compress and allow the patient to breathe fresh air for a time.

8. When respiration is arrested, seize the tongue with the forceps and draw it out, and immediately commence artificial respiration. If the respiration is not established after a few seconds, place the head low, forcibly flagellate the cheeks, keep the tongue out, and continue the artificial respiration for five, ten, fifteen or even twenty minutes, if necessary.

9. When the respiration is noisy, pass into the back of the throat a sponge mounted on a forceps, in order to remove the mucosities existing there, as they frequently do in patients suffering from colds.

10. There is but one contra-indication to the employment of chloroform—namely, advanced phthisis. Affections of the heart are not contra-indications.

11. Hysterical subjects should be distrusted.

12. Alcoholic subjects are very long and difficult in being brought under the influence of chloroform, but they may take it without danger.—*Medical Times and Gazette.*

